# Supplementary material for: Splice-Junction-Based Mapping of Alternative Isoforms in the Human Proteome
Source: Cell Rep. Author manuscript; Available in PMC 2020 Jan 15. (PMC6961840; doi:10.1016/j.celrep.2019.11.026)
Supplement: 2 [file NIHMS1546469-supplement-2.zip › DF1/PXD010154/Testis/Testis_1_ZFPM1_ELEGQEPEPRPTEEEPGSPWSGPEPSPDPAAGGR.pdf]

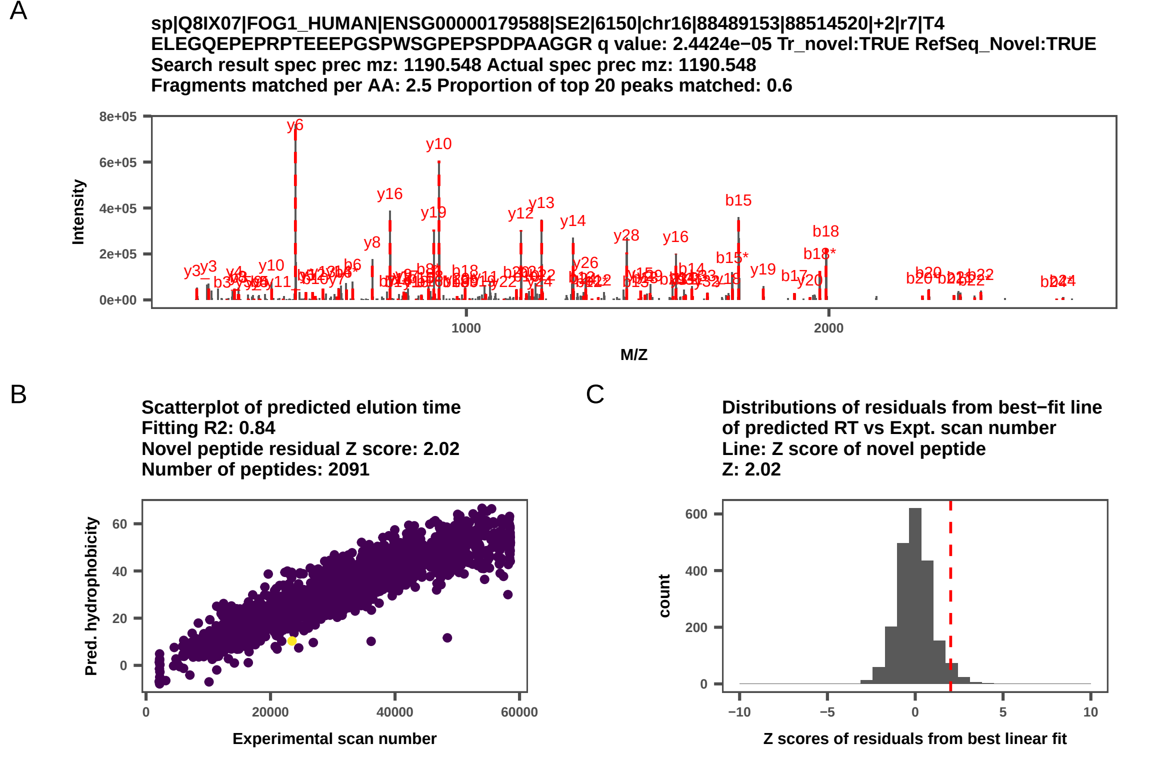
A
sp|Q8IX07|FOG1_HUMAN|ENSG00000179588|SE2|6150|chr16|88489153|88514520|+2|r7|T4
ELEGQEPEPRPTEEEPGSPWSGPEPSPDPAAGGR q value: 2.4424e−05 Tr_novel:TRUE RefSeq_Novel:TRUE
Search result spec prec mz: 1190.548 Actual spec prec mz: 1190.548
Fragments matched per AA: 2.5 Proportion of top 20 peaks matched: 0.6
Intensity
M/Z
B
Scatterplot of predicted elution time
Fitting R2: 0.84
Novel peptide residual Z score: 2.02
Number of peptides: 2091
Pred. hydrophobicity
Experimental scan number
C
Distributions of residuals from best−fit line
of predicted RT vs Expt. scan number
Line: Z score of novel peptide
Z: 2.02
count
Z scores of residuals from best linear fit
